# Sex-specific associations of sex hormone binding globulin and risk of bladder cancer

**DOI:** 10.1515/med-2025-1163

**Published:** 2025-02-28

**Authors:** Junyong Ou, Hai Bi, Haobin Zhou, Zhanyi Zhang, Peichen Duan, Haoming Yin, Zezhen Zhou, Zhixin Xie, Xiaojun Tian, Jianfei Ye, Shudong Zhang

**Affiliations:** Department of Urology, Peking University Third Hospital, Peking University Health Science Center, Beijing, 100191, China; Department of Urology, Shanghai General Hospital, Shanghai Jiao Tong University School of Medicine, Shanghai, 200080, China; The First Clinical College of Guangzhou Medical University, Guangzhou, Guangdong, 511436, China; The Second Clinical College of Guangzhou Medical University, Guangzhou, Guangdong, 511436, China; Department of Urology, Peking University Third Hospital, Peking University Health Science Center, 49 North Garden Road, Beijing, 100191, China

**Keywords:** bladder cancer, sex hormone binding globulin, risk, testosterone, genome-wide association studies

## Abstract

**Background:**

Males have a three times higher risk of a diagnosis of bladder cancer (Bca) than females. Sex hormone-binding globulin (SHBG) may be associated with Bca risk. However, the sex-specific role of SHBG in Bca remains unclear. In this study, we aimed to determine the role of SHBG in Bca.

**Methods:**

A sex-specific univariable Mendelian randomization (MR) analysis involving 369,426 men and 395,375 women was conducted to assess the causal relationship between SHBG and testosterone and Bca risk. Sensitivity analyses and multivariable MR were conducted to confirm the robustness of our results. Linkage disequilibrium score regression assessed the genetic correlation between these diseases influenced by heredity.

**Results:**

Univariable MR results showed that one-SD elevated SHBG was related to a low risk of Bca in males (OR: 0.60, 95% CI: 0.39–0.93; *p* = 0.022) but had no benefit in females. Genetically predicted BT was positively associated with Bca risk in males (OR: 1.59; 95% CI: 1.06–2.40; *p*  =  0.027). In multivariable MR, higher SHBG levels were not related to male Bca risk after controlling for BT.

**Conclusions:**

Our findings do not provide evidence to support a causal relationship between SHBG and Bca risk in males although an association was observed in the univariable analysis. Further research is needed to identify the underlying pathways.

## 1 Introduction

Bladder cancer (Bca) is a common malignant tumor of the urinary system. It is a major economic burden on the healthcare system and is well known to be associated with sex bias [1]. Sex differences in Bca incidence have been observed in epidemiological and clinical studies, with a male-to-female risk ratio of 3:1 in terms of diagnosis [2,3]. Although smoking and exposure to occupational carcinogens are factors that contribute to the sex-dependent differences in Bca incidence, Bca still occurs in males after controlling exposure to these carcinogenic factors [4–6]. Therefore, intrinsic factors are likely to play key roles in urothelial carcinogenesis. Experimental and clinical evidence suggests the involvement of androgens and androgen receptors (AR) [7,8]. However, not all studies have reached unanimous conclusions.

Sex hormones and their receptors have been posited as potential contributory factors responsible for sex-based disparities in Bca incidence [9]. Androgens are likely to contribute to sexual dimorphism in Bca by directly and indirectly influencing various cellular processes, such as the synthesis of cytokines, growth factors, and vasoactive substances. Sex hormone-binding globulin (SHBG) is involved in binding to sex hormones and regulating their biological activity [2]. Nevertheless, establishing these associations conclusively through randomized controlled trials poses considerable challenges. Observational studies face difficulties in distinguishing the effects of SHBG levels from those attributable to sex hormones, given their intricate interrelationship. Furthermore, there is a lack of research investigating the sex-specific roles of SHBG in Bca.

To address the lack of experimental evidence for SHBG involvement in Bca, we used a Mendelian randomization (MR) study design, using naturally occurring genetic variants that affect SHBG levels throughout life [10]. Genetic variants are determined at conception; therefore, using this approach establishes the role of SHBG in Bca, excluding the potential confounding effects of socioeconomic position or other factors. This research utilized linkage disequilibrium score regression (LDSC) analysis of GWAS summary statistics to investigate the genetic associations among SHBG, BT, and Bca, with a focus on hereditary contributions. We conducted a sex-specific MR analysis using published single nucleotide polymorphisms (SNPs) to predict SHBG in both males and females and controlled for bioavailable testosterone (BT) levels.

## 2 Materials and methods

### 2.1 Genetic correlation analysis

LDSC is an effective method for analyzing genetic correlations [11]. We employed LDSC to examine the shared polygenic structure between diseases, complementing MR analysis. Linkage disequilibrium (LD) scores and weights for the European population were precomputed from 1,000 Genomes data by LDSC’s original developers. The analysis utilized the R package “ldscr.”

### 2.2 Research design

**Figure 1:**
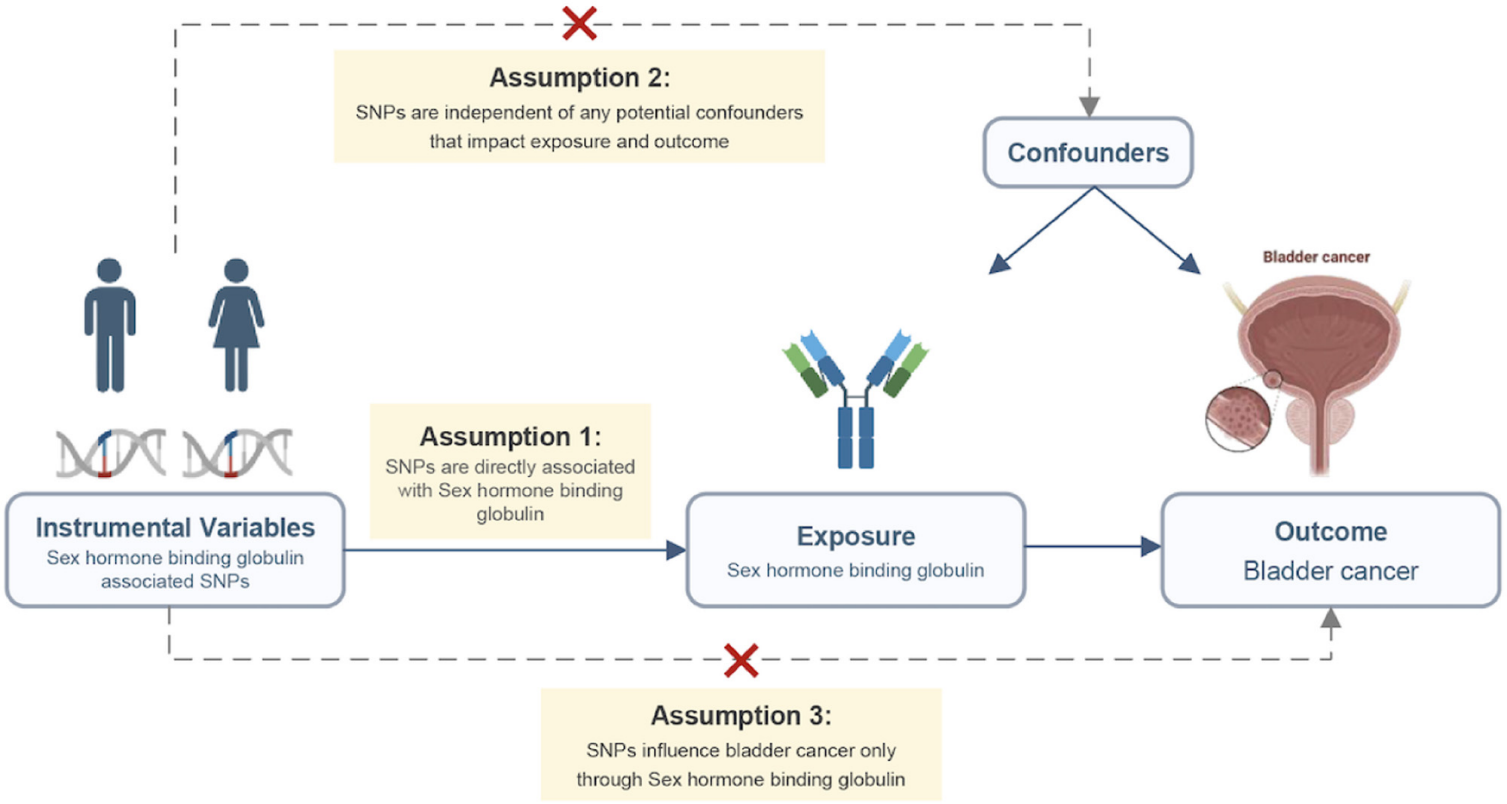
Study design. Bca: bladder cancer; SHBG: sex hormone-binding globulin.

Initially, we employed univariate MR to evaluate the overall influence of SHBG and BT on Bca risk. Subsequently, we conducted a multivariable MR analysis to examine the direct and independent effects of these traits on the observed outcomes. This study followed the MR guidelines based on three key assumptions: a close relationship between genetic markers and SHBG, the independence of instrumental variables (IVs) from confounding factors, and the exclusive effect of IVs on Bca through SHBG [12] (Figure 1). The data utilized in this study were derived from publicly available genome-wide association studies (GWAS), which have been granted ethical approval and informed consent.

### 2.3 Data sources

#### 2.3.1 Selection of genetic variants associated with exposure

In order to mitigate bias stemming from sample overlap, we utilized nonoverlapping datasets for each exposure-outcome pair. We selected SNPs from GWASs in the UK Biobank to serve as IVs for SHBG (GWAS ID: ieu-b-4871 for males, ieu-b-4870 for females) and BT (GWAS ID: ieu-b-4868 for males, ieu-b-4869 for females) of European ancestry [13]. SHBG genetic variations were derived from the UK Biobank’s summary sex-specific GWAS (185,221 white British males and 214,989 white British females). BT genetic variations were derived from the UK Biobank’s summary sex-specific GWAS (184,205 white British males and 180,386 white British females). We examined SNPs with genome-wide significance (*p* < 5 × 10^−8^) and utilized LD analysis to eliminate SNPs with mutual LD above the limit (LD clumping panel kb = 5,000, *R* ^2^ < 0.01) [14].

To address potential pleiotropy, we chose variables known to be involved with Bca. For instance, body mass index (BMI) might be a driver [15] or downstream factor [13] of SHBG. We used the PhenoScannerV2 (www.phenoscanner.medschl.ca.ac.uk) to exclude confounders, such as BMI, smoking, long-term exposure to industrial chemicals, arsenic contamination, chemotherapy drugs, such as cyclophosphamide, or pioglitazone for type 2 diabetes [5,16,17]. Finally, we included 48 and 66 SNPs as IVs for SHBG in males and females, respectively, and 53 and 77 SNPs as IVs for BT in males and females, respectively. To validate the strength of the identified SNPs, we produced *F*-statistics and conditional *F*-statistics for univariate and multivariable MR analyses.

#### 2.3.2 Genetic variants associated with Bca

Summary data on the sex-specific genetic variants of Bca were obtained from FinnGen R10 comprising 175,121 European individuals (1,115 Bca cases and 174,006 controls) [18]. The GWAS analyses were adjusted for principal components.

#### 2.3.3 Genetic variants for multivariable MR

We employed multivariable MR analysis to control for BT since SHBG was strongly related to this particular variable [13]. Genetic predictors of SHBG and BT were obtained from the UK Biobank. We excluded duplicate SNPs and those with a high correlation (*r* ^2^ > 0.05) using LDLink. The remaining SNPs were used for multivariable MR analysis.

#### 2.3.4 Statistical analysis

The primary MR approach was inverse variance weighting (IVW), employed to assess the effect of a one-standard deviation (SD) increment in genetically predicted exposure on the outcome, which was reported as odds ratio (OR) accompanied by a 95% confidence interval (CI). Other MR methods, such as MR-Egger, weighted median, and weighted mode, were also used to investigate the consistency of effect estimates.

As a first step, SNPs with a genome-wide significance level of *p* < 5 × 10^−8^ and an *F*-statistic >10 were carefully selected. To address potential confounding factors, we utilized the PhenoScannerV2 website to exclude genetic confounders associated with Bca. For the assessment of heterogeneity and horizontal pleiotropy, Cochran’s *Q* statistic was employed to quantify heterogeneity, while radial MR was utilized to eliminate outliers [19]. Additionally, MR-Egger regression and the MR-Presso method were employed to evaluate the influence of horizontal pleiotropy upon effect estimations. Sensitivity analysis was conducted through leave-one-out tests to assess the effects of individual variants on the observed associations. For multivariable MR, the test for heterogeneity and pleiotropy were similar to those described above for univariate MR [20].

R2 indicates the proportion of SHBG variation that may be attributed to SNPs.

Furthermore, the required sample size for Bca events was estimated using the log OR, which measures the patient-to-non-patient ratio. All MR analyses were performed using the two-sample MR and MR packages in R (version 4.1.0).

## 3 Results

### 3.1 Genetic correlation analysis

The results of the genetic correlation analysis are presented in Table 1. We estimated a positive genetic correlation between the following pairs of diseases: SHBG (male) and Bca (male) (rg = 0.663, *p*-value = 1.52 × 10^−05^), SHBG (female) and Bca (female) (rg = 0.697, *p*-value = 7.95 × 10^−58^), and BT (male), and Bca (male) (rg = 0.563, *p*-value = 1.12 × 10^−39^).

**Table 1:** Results of genetic correlation analysis

Trait1	Trait2	Genetic correlation	Standard error	*p*-value
SHBG (male)	Bca (male)	0.663	0.144	1.52 × 10^−5^
SHBG (female)	Bca (female)	0.697	0.043	7.95 × 10^−58^
BT (male)	Bca (male)	0.563	0.043	1.12 × 10^−39^
BT (female)	Bca (female)	−0.286	0.202	0.156

SHBG: sex hormone-binding globulin; BT: bioavailable testosterone; Bca: bladder cancer.

### 3.2 IVs

In the univariate MR analysis, we employed 48 and 66 genome-wide significant SNPs previously reported in males and females, respectively, for the assessment of SHBG. Furthermore, 53 and 77 genome-wide significant SNPs previously identified in males and females, respectively, were utilized for investigating BT. Following the methodology outlined by Burgess et al., all SNPs had an *F-*statistic exceeding >10, indicating the absence of weak instruments in univariate MR analysis (Table S1).

### 3.3 Causal effect of SHBG on Bca using univariable MR

**Figure 2:**
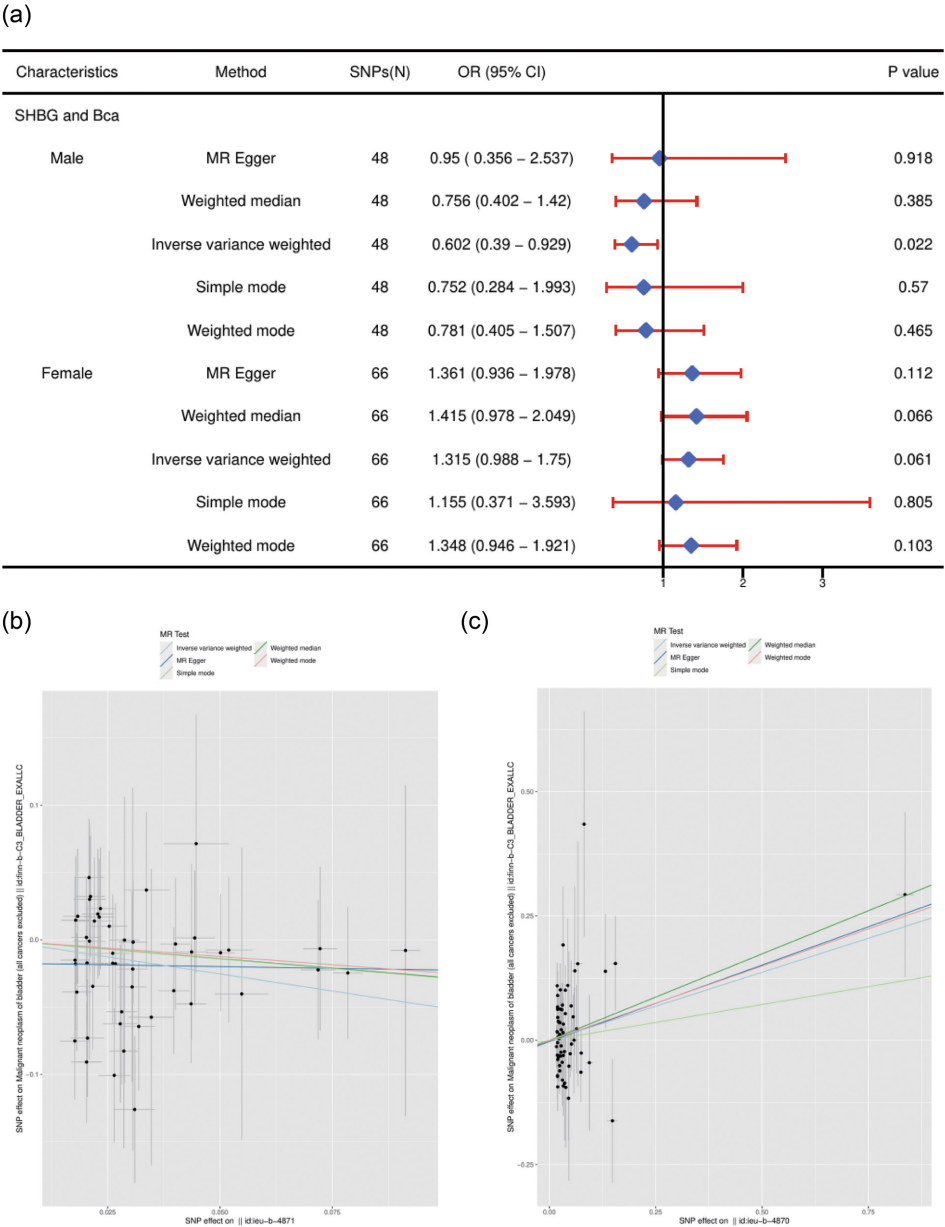
Univariable MR results of SHBG using different methods. (a) The combined forest plot of SHBG on Bca. (b) The scatter plots of SHBG on Bca in males. (c) The scatter plots of SHBG on Bca in females. The number of genetic variants, OR, 95% CI, *p* values, and MR methods of associations are contained. SNPs(N): the number of single-nucleotide polymorphisms used as IVs; OR: the combined causal effect; CI: confidence interval; SHBG: sex hormone binding globulin; Bca: bladder cancer.

The random-effects IVW approach was the main analytical method employed. In two sample MR, there was no indication of instrumental heterogeneity (Cochran’s *Q* test, *p* > 0.05). One SD elevated SHBG was related to a low risk of Bca in males OR: 0.60, 95% CI: 0.39–0.93; *p* = 0.022; Bonferroni’s correction [*p* = 0.05/2]). The causal effect estimation of the MR-Egger test and other methods were similar in direction and magnitude. However, genetically predicted SHBG had no association with Bca in females (OR: 1.32, 95% CI: 0.99–1.75 *p* = 0.061) (Figure 2).

**Figure 3:**
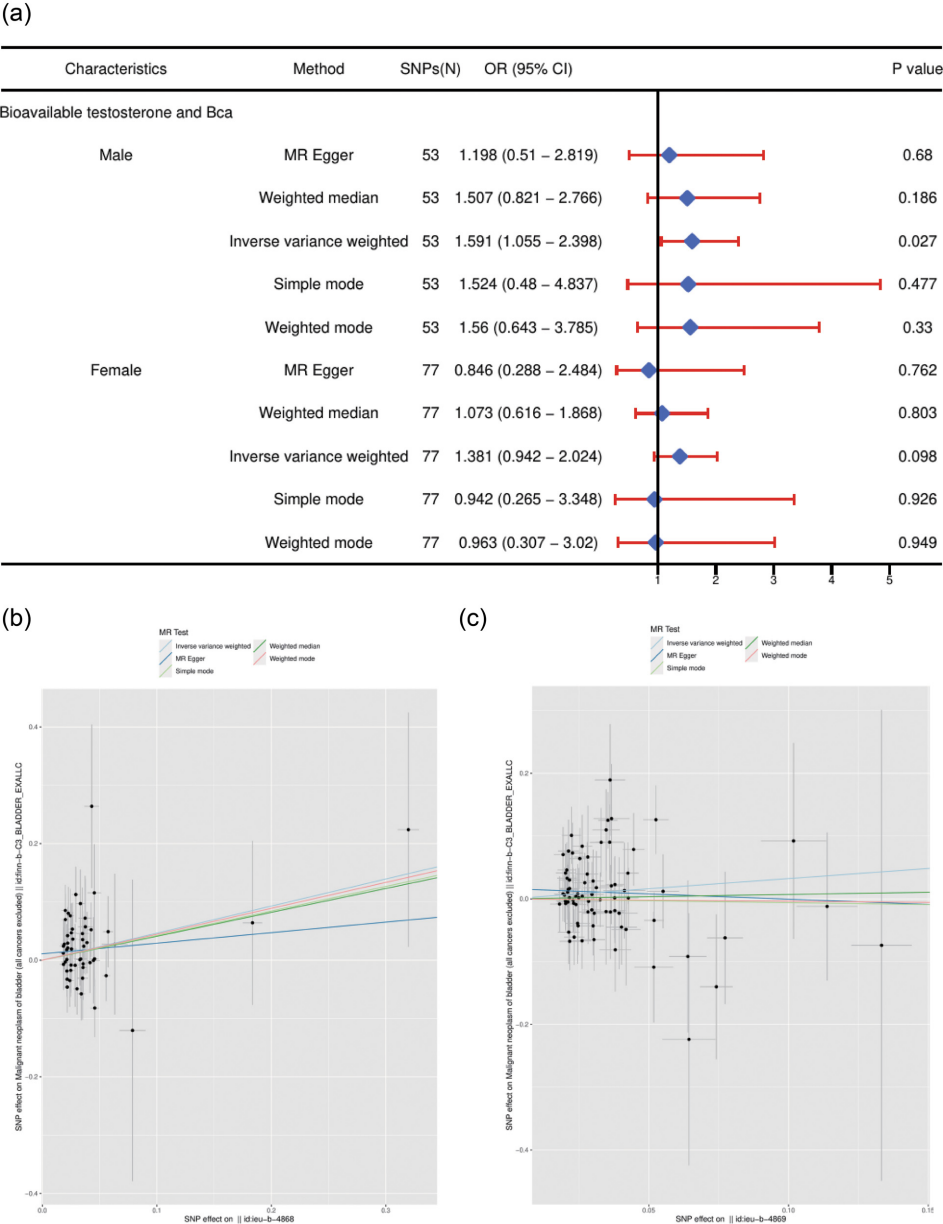
Univariable MR results of BT using different methods. (a) The combined forest plot of BT on Bca. (b) The scatter plots of BT on Bca in males. (c) The scatter plots of BT on Bca in females. The number of genetic variants, OR, 95% CI, *p* values, and MR methods of associations are contained. SNPs(N): the number of single-nucleotide polymorphisms used as IVs; OR: the combined causal effect; CI: confidence interval; Bca: bladder cancer.

One SD increase in BT was associated with a significantly increased Bca risk in males (OR: 1.59; 95% CI: 1.06–2.40; *p*  =  0.027). However, the result did not reach statistical significance after Bonferroni’s correction (*p* = 0.05/2), indicating a suggestive causal association. MR analyses, such as MR-Egger, weighted median, and weighted mode, were similar in direction and magnitude. In females, genetically predicted BT was unrelated to Bca (OR: 1.38, 95% CI: 0.94–2.02; *p* = 0.098) (Figure 3). The scatter plots of univariable MR analyses performed on FinnGen data using different methods showing the effect of exposure on Bca in male and female. The single MR effect of univariable MR analyses showing the effect of exposure on Bca is shown in Figure S1.

The MR-Egger intercept test was employed to assess pleiotropy. There was no directional pleiotropy found using MR-Egger regression or MR-PRESSO analysis (Table 2). The results of a leave-one-out sensitivity analysis demonstrated that no one SNP was responsible for the overall impact of sex hormones on Bca (Figure S2). The MR funnel diagram was symmetrical (Figure S3).

**Table 2:** Sensitive analyses result in univariable MR for Bca

Outcome	Exposure	IVW- *Q* test	MR-Egger			MR-Presso Global test
		*p*-value	Intercept	SE	*p*-value	*p*-value
Bca						
Male	SHBG	0.987	−0.017	0.017	0.317	0.994
	BT	0.849	0.011	0.015	0.463	0.816
Female	SHBG	0.250	−0.003	0.009	0.779	0.312
	BT	0.663	0.016	0.017	0.343	0.576

IVW: inverse-variance weighted; SE: standard error; SHBG: sex hormone-binding globulin; BT: bioavailable testosterone; Bca: bladder cancer.

### 3.4 Causal effect of SHBG on Bca using multivariable MR

**Figure 4:**
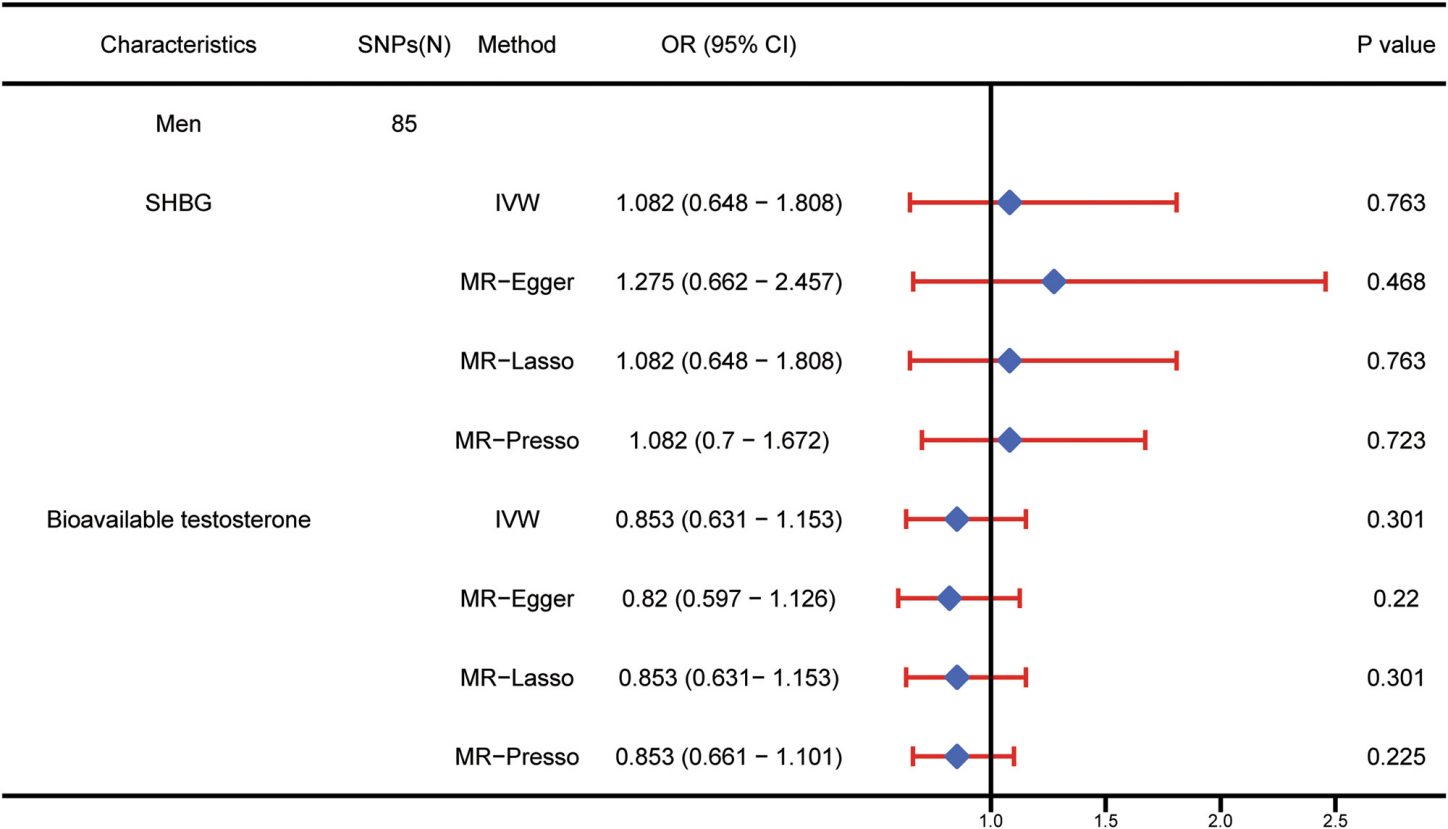
Multivariable MR results of SHBG and BT. The number of genetic variants, OR, 95% CI, *p* values, and MR methods of associations are contained. SNPs(N), the number of single-nucleotide polymorphisms used as IVs; OR, the combined causal effect; CI, confidence interval; *p* value, *p* value of the causal estimate; SHBG: sex hormone-binding globulin; Bca: bladder cancer; IVW: inverse variance weighted.

After adjusting for BT in the multivariable MR for males, genetically predicted increased SHBG was not associated with the risk of Bca (OR: 1.08; 95% CI: 0.65–1.81; *p*  =  0.763). After controlling for SHBG, the causal relationship between BT and Bca did not remain statistically significant (OR: 0.85; 95% CI: 0.63–1.15; *p*  =  0.301) (Figure 4). The conditional *F*-statistics for the SHBG and BT were 40.2 and 95.0%, respectively. The conditional *F*-statistics indicated the absence of weak instruments in multivariable MR. Cochran’s *Q* statistic testing indicated the absence of heterogeneity in multivariable MR. Horizontal pleiotropy was not observed (Table 3). In direction and magnitude, the estimations were stable to the multivariable in MR-Egger, MR-Lasso, and MR-Presso approaches.

**Table 3:** Conditional *F*-statistics and sensitive analyses result in multivariable MR for Bca in males

Outcome	Exposure	*F*-statistics	*Q*-test		MR-Egger			MR-Presso Global test
			*Q*-statistic	*Q*_*p*	Intercept	SE	*p*-value	*p*-value
Bca								
	SHBG	40.177	59.574	0.976	−0.006	0.008	0.431	0.977
	BT	95.046						

SE: standard error; SHBG: sex hormone-binding globulin; BT: bioavailable testosterone; Bca: bladder cancer.

## 4 Discussion

Understanding the influence of sex differences on the incidence, prevalence, and severity of Bca has become an increasingly important area of research. Sex should be regarded as a crucial biological variable to be included in future Bca research [21]. To the best of our knowledge, this study is the first to examine the sex-specific risk of Bca from a genetic perspective. We investigated the relationship between SHBG and Bca using univariate and multivariable MR analyses.

The glycoprotein SHBG is predominantly synthesized in the liver and exists as a homodimeric protein with a molecular weight of 90–100 kDa. Its binding ability extends to all androgens and estrogens, except for dehydroepiandrosterone sulfate and androstenedione. The free hormone hypothesis suggests that because binding proteins are the gatekeepers of steroid action, the biological activity of a hormone is best represented by the concentration of the free hormone rather than its total concentration. Notably, SHBG contributes considerably to the balance between free and protein-bound components of plasma and serves as a transporter of steroid hormones, facilitating their transport from the point of synthesis to their target location. Particularly for androgens, interactions with SHBG and albumin determine the delicate balance between bioavailability and total testosterone, thereby controlling tissue exposure. This balance can be influenced by factors such as aging, genetics, and various pathological conditions that affect target tissue hormone exposure [22]. Although the precise mechanism is yet unknown, it is conceivable that the regulatory function of SHBG in modulating the levels of free/BT in the bloodstream contributes to this protective association.

Additionally, SHBG may play a role in male Bca through other pathways. The development of Bca is influenced by inflammation in several ways. Chronic inflammation can cause DNA damage and mutations in bladder epithelial cells, thereby increasing the risk of Bca [23]. Bca cells can trigger an “inflammatory cytokine storm,” stimulating the secretion of tumor growth-promoting factors and weakening the cytotoxic function of immune cells. Cytokines and growth factors released by these cells may stimulate Bca cell proliferation [24]. Increased levels of reactive oxygen species, lipid peroxidation products, pro-inflammatory cytokines, and pro-angiogenic factors may induce inflammatory responses, ultimately activating Bca angiogenesis [25]. SHBG inhibits inflammation *in vitro*, which is not altered by co-supplementation with testosterone or estradiol [26], supporting a pathway through inflammation. Further investigation into potential pathways, especially those associated with sex-specific responses to SHBG and Bca, would be beneficial. Similarly, a meta-analysis of previous clinical trials showed that androgen deprivation therapy (ADT) may have antitumor effects on Bca [8]. However, these findings should be interpreted with caution. The relationship between ADT and Bca in previous observational studies has been inconsistent, and clinical trials to validate their efficacy are still lacking [7]. The efficacy of abiraterone and other approved anticancer agents for urothelial cancer in the treatment of Bca may be further elucidated through an ongoing clinical trial [27].

This study design has several strengths that contribute to the validity of our findings. This study adheres strictly to the assumptions of MR so that potential confounding factors and reverse causality are minimized, and an independent correlation between SHBG and Bca risk can be established. Moreover, LDSC aids in distinguishing true polygenic effects from confounding factors such as implicit associations and population stratification. Notably, this study is the first to comprehensively and systematically analyze prospective studies to evaluate the role of SHBG in Bca risk. We also employed MR-Egger, which provides robustness against pleiotropy, a potential limitation of MR. Replication with larger samples is required in such cases. Furthermore, the high *F*-statistics of the genetic instruments employed in this study mitigates concerns regarding the presence of weak instruments that could potentially introduce bias into our findings.

Despite its novelty, this study has several limitations. First, the research might have been influenced by survivorship bias and competing risks, which preclude the occurrence of Bca. We adjusted the common confounders of Bca. Second, compensatory processes or feedback mechanisms may dilute genetic effects, resulting in skewed MR estimations. However, this did not explain the observed beneficial association between SHBG and Bca. A common limitation is that non-muscle-invasive Bca is not distinguished from muscle-invasive disease. We could not stratify Bca across diagnostic types. Third, it is crucial to recognize a fundamental limitation of LDSC: while it indicates overall positive, negative, or absent genetic correlations, it does not account for mixed directional effects among shared genetic variants. We agree that the potential pleiotropic nature of the included variants and the shared genetic risk between SHBG and BT could distort the results. Consequently, additional research is needed to provide a more detailed quantification of polygenic overlap and deepen insights into the genetic interconnections between these diseases [28].

Regardless of the consistent and beneficial associations observed in this study, caution should be exercised when interpreting our findings [29]. Instead of focusing on the immediate results of an exogenous exposure, MR considers the long-term consequences of an endogenous one. Consequently, the impact on male Bca may not be significant in terms of factors that regulate SHBG.

Our findings do not provide evidence to support a causal relationship between SHBG and Bca risk in males. While an association was observed in the univariable analysis, this is likely influenced by shared genetic risk variants and the pleiotropic effects of the included variants. Further studies, including formal mediation analyses, are required to explore the potential interplay between SHBG, BT, and Bca risk.

## Abbreviations

ADT: androgen deprivation therapy
AR: androgen receptor
Bca: bladder cancer
BMI: body mass index
CI: confidence interval
GWAS: genome-wide association study
IVs: instrumental variables
IVW: inverse variance-weighted
MR: Mendelian randomization
OR: odds ratio
SHBG: sex hormone-binding globulin
SNP: single nucleotide polymorphism

## Supplementary Material

Supplementary material
