# Supplementary material for: Sex-specific associations of sex hormone binding globulin and risk of bladder cancer
Source: Open Med (Wars). 2025 Feb 28;20(1):20251163. doi: 10.1515/med-2025-1163 (PMC11889508; doi:10.1515/med-2025-1163)
Supplement: Supplementary material [file med-2025-1163-suppl.pdf]

Supplementary material

Table S1: The quantity of instrumental variables and phenotypic variance

Outcome	Male SHBG	Male Bioavailable testosterone	Female SHBG	Female Bioavailable testosterone
**Bca**				
SNPs	48	53	66	77
R^2%	2.7	2.6	3.3	2.6
F statistics	105.0	93.5	109.7	63.7

SHBG: sex hormone binding globulin; Bca: bladder cancer; SNPs: single nucleotide polymorphisms; R^2 is the percentage of phenotypic variance that single-nucleotide polymorphisms (SNPs) account for, and it can be calculated using the “get r pn” function in the “TwoSampleMR” R package.

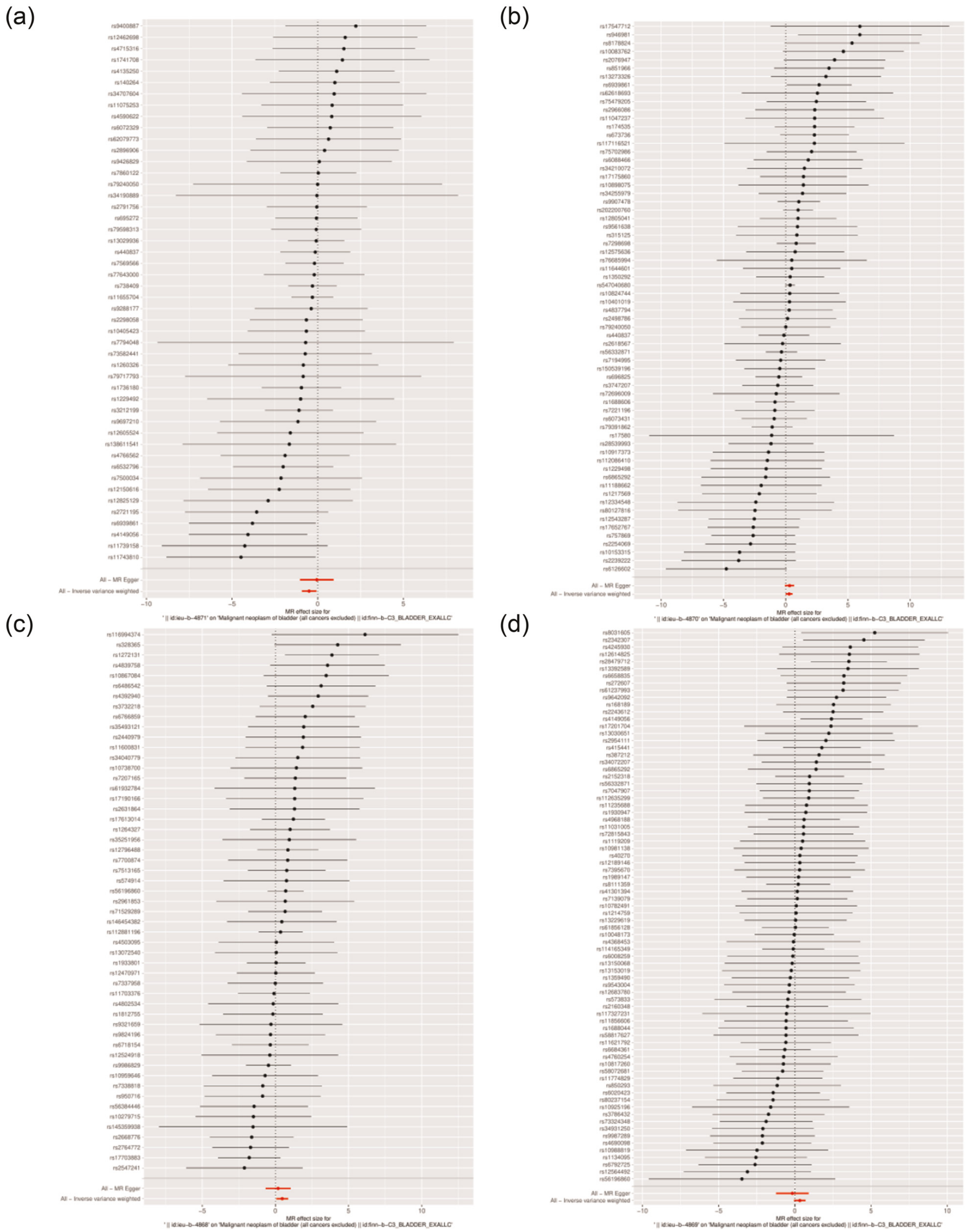

Figure S1: Single MR effect of univariable MR analyses showing the effect of exposure on the Bca in male and female via different methods. (a)–(d) represent the effect of SHBG on Bca in male, SHBG on Bca in female, bioavailable testosterone on Bca in male and bioavailable testosterone on Bca in female, respectively.

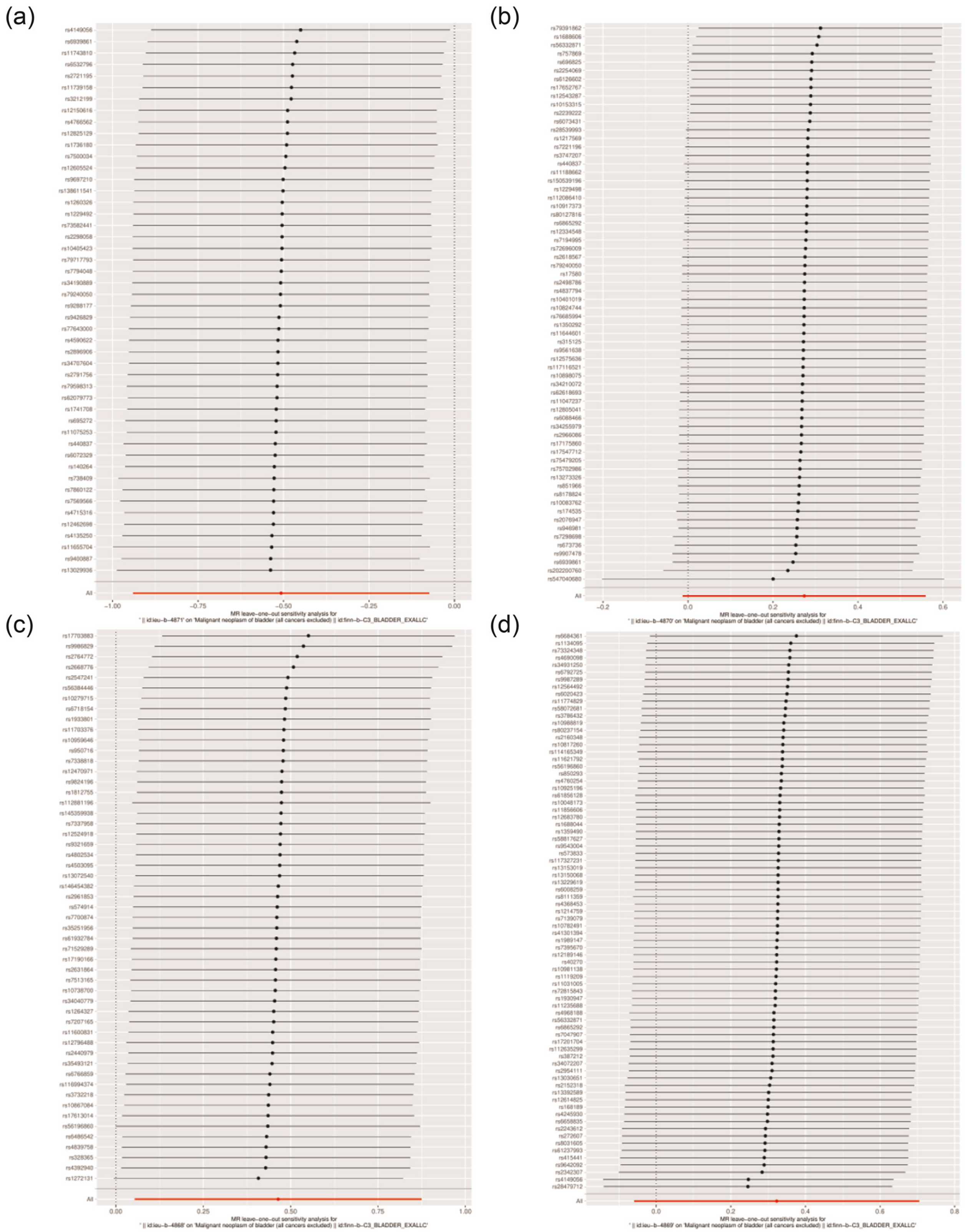

Figure S2: Leave-one-out analysis of univariable MR analyses. (a)–(d) represent the effect of SHBG on Bca in male, SHBG on Bca in female, bioavailable testosterone on Bca in male and bioavailable testosterone on Bca in female, respectively.

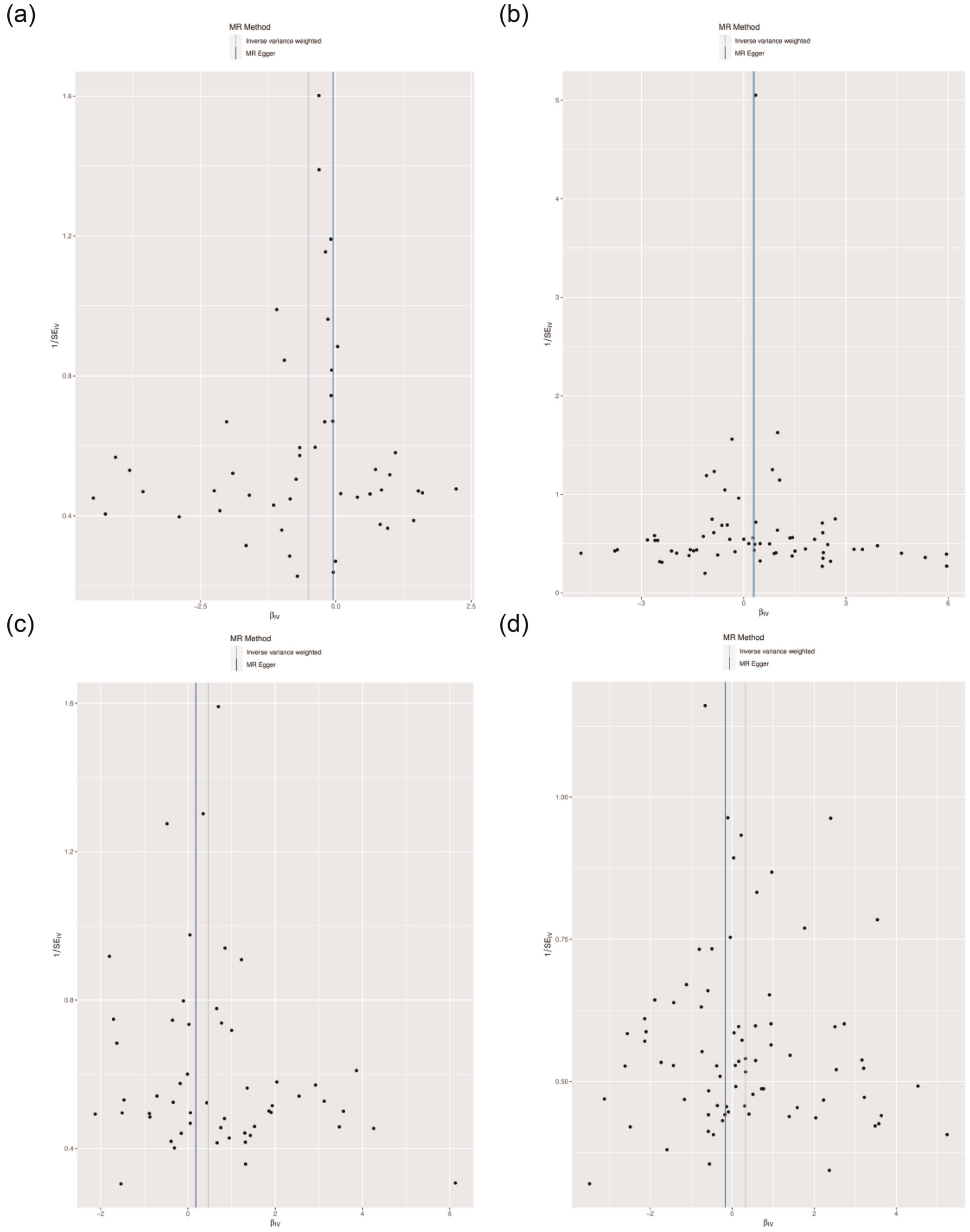

Figure S3: Funnel plot analysis of univariable MR analyses. (a)–(d) represent the effect of SHBG on Bca in male, SHBG on Bca in female, bioavailable testosterone on Bca in male and bioavailable testosterone on Bca in female, respectively.
